# Chimeric Antigen Receptors T Cell Therapy in Solid Tumor: Challenges and Clinical Applications

**DOI:** 10.3389/fimmu.2017.01850

**Published:** 2017-12-22

**Authors:** Hamid R. Mirzaei, Analiz Rodriguez, Jennifer Shepphird, Christine E. Brown, Behnam Badie

**Affiliations:** ^1^Department of Medical Immunology, School of Medicine, Tehran University of Medical Sciences, Tehran, Iran; ^2^Division of Neurosurgery, Department of Surgery, City of Hope National Medical Center, Duarte, CA, United States; ^3^Department of Hematology and Hematopoietic Cell Transplantation, T Cell Therapeutics Research Laboratory, City of Hope Beckman Research Institute, Duarte, CA, United States

**Keywords:** immunotherapy, T cell therapy, chimeric antigen receptor, CAR, solid tumors

## Abstract

Adoptive cellular immunotherapy (ACT) employing engineered T lymphocytes expressing chimeric antigen receptors (CARs) has demonstrated promising antitumor effects in advanced hematologic cancers, such as relapsed or refractory acute lymphoblastic leukemia, chronic lymphocytic leukemia, and non-Hodgkin lymphoma, supporting the translation of ACT to non-hematological malignancies. Although CAR T cell therapy has made remarkable strides in the treatment of patients with certain hematological cancers, in solid tumors success has been limited likely due to heterogeneous antigen expression, immunosuppressive networks in the tumor microenvironment limiting CAR T cell function and persistence, and suboptimal trafficking to solid tumors. Here, we outline specific approaches to overcome barriers to CAR T cell effectiveness in the context of the tumor microenvironment and offer our perspective on how expanding the use of CAR T cells in solid tumors may require modifications in CAR T cell design. We anticipate these modifications will further expand CAR T cell therapy in clinical practice.

## Introduction

For many years, the cornerstones of cancer treatment have been surgery, chemotherapy, and radiation, and more recently targeted therapies. Although these approaches have contributed to improved outcomes, most malignancies still carry a poor prognosis. Targeted anticancer approaches provide individualized therapy to combat the complexity of most malignancies and increase the probability of success. Currently, interest is increasing in immunotherapies, which harness the power of a patient’s immune system to fight disease. One approach to cancer immunotherapy entails genetically engineering a patient’s T cells to express chimeric antigen receptors (CARs) that recognize and attack tumor cells. The CAR consists of an antibody or ligand-derived targeting ectodomain fused with a hinge, a trans-membrane domain, and intracellular T cell signaling domains. When expressed by a T cell, CARs confer antigen specificity determined by the targeting domain (1, 2). In contrast to conventional T cell receptors (TCRs), which recognize antigens in a major histocompatibility complex (MHC)-dependent manner, CARs can potentially redirect the effector functions of a T cell toward any protein or non-protein target expressed on the cell surface. This strategy thereby avoids the requirement of antigen processing and presentation by the target cell and is applicable to non-classical T cell targets like carbohydrates (3). Circumventing human MHC-restriction renders the CAR T cell approach as a universal treatment, broadening the potential applicability of adoptive T cell therapy.

Four generations of CAR are being investigated in preclinical and ongoing clinical studies. The CAR “generation” typically refers to the intracellular signaling domains incorporated in the receptor molecule. First-generation CARs include only CD3ζ as an intracellular signaling domain; second-generation CARs include in addition to CD3ζ, a single co-stimulatory domain, such as CD28, 4-1BB (CD137), CD27, or OX40; third-generation CARs contain CD3ζ and two co-stimulatory domains, such as CD28, 4-1BB, or other co-stimulatory molecules. CARs may be further manipulated through the introduction of additional genes, including those encoding potent antitumor cytokines (e.g., IL-12 and Il-15) or co-stimulatory ligands (e.g., 4-1BBL), thus producing “armored” fourth-generation CAR T cells (4, 5).

Chimeric antigen receptors targeting the B cell receptor-associated protein CD19, developed for the treatment of B cell leukemia and lymphomas, have been the most clinically tested to date. Exciting progress with CD19-CAR T cell therapy across multiple institutions employing different therapeutic designs has led to the successful commercialization of this adoptive immunotherapy. In August 2017, the US Food and Drug Administration (FDA) approved the first CAR T cell therapy, tisagenlecleucel (Kymriah, Novartis Pharmaceuticals Corp.), for the treatment of certain pediatric and young adult patients with B cell acute lymphoblastic leukemia. This CAR T cell therapy has achieved remarkable outcomes in children and young adults with relapsed and often refractory disease, with complete response (CR) rates of 70–90% (6). Soon after the first approval, in October 2017, the FDA approved the CAR T cell therapy axicabtagene ciloleucel (Yescarta, Kite Pharma, Inc.) for the treatment of adult patients with certain types of B cell lymphoma. In lymphomas and other B cell malignancies, CAR T cell therapy, while effective, has shown lower CR rates, near 55% (6). This highlights the impact of tumor-specific parameters on the effectiveness of CAR T cells. Both CARs specifically bind CD19, an antigen that works well as a target for hematological malignancies because it is nearly uniformly expressed on malignant cells, save for conditions of therapeutic selective pressure, where antigen loss has been observed (7). Because CD19 appears on all B cells, both healthy and malignant, CD19-CAR T cell treatment may cause B cell aplasia, but the condition can be managed with intravenous immunoglobulins and close infection monitoring.

Despite progress in the treatment of hematological cancers with CAR T cells, treatment of solid tumors has proven more difficult. Here, we review CAR T cell therapy in solid tumors and discuss challenges and corresponding strategies to overcome them.

## Challenges for CAR T Cell Therapy in Solid Tumors

The limited success of CAR T cell therapy against solid tumors may be due to many factors, including: (i) the lack of a unique tumor-associated antigen (TAA) in most cancers; (ii) the inability of *ex vivo* expanded CAR T cells to persist and proliferate following adoptive transfer; (iii) inefficient trafficking of CAR T cells to tumor sites; (iv) heterogeneous expression of the targeted antigen(s) leading to outgrowth of antigen-negative tumor variants; (v) the lack of survival and growth factors (e.g., IL-2); (vi) the presence of immunosuppressive molecules and cells; and (vii) the metabolically hostile tumor microenvironment. Table 1 lists several fundamental characteristics of solid tumors that present obstacles to CAR T cell therapy.

**Table 1:** Challenges for chimeric antigen receptor (CAR) T cell therapy in solid tumors.

Challenge(s)		Overcoming strategy(s)	Reference
Tumor microenvironment	Soluble molecules	Use of gene edited CAR T cells that disrupt sensitivity to inhibitory pathways such as adenosine and prostaglandin E2 signaling, PD-1, IDO, and TIM-3 inhibitory molecules	(8–13)

	Immunosuppressive immune cells	The concomitant application of CAR T cells with blockage and depletion of various immunosuppressive molecules and cells such as Tregs and myeloid-derived suppressor cells	(10, 14–16)
		Use of armored-CAR T cells	

	Physical and metabolic barriers	Generation of CAR T cells which degrade the extracellular matrix and target tumor-associated stromal cells to facilitate infiltration of T cells into solid tumor masses	(17, 18)

Trafficking		Use of CAR T cells overexpressing chemokine receptors or combined application of CAR T cells with an oncolytic virus armed with the chemokines that match the chemokines receptors expressed by T cells	(19–22)
		Genetic addition of molecules which improve CAR T localization	
		Local delivery of CAR T cells	

Target antigen heterogeneity		Use of CARs targeting multiple antigens	(18, 23, 24)
		Use of dual-specific T cells	
		Monitoring of patients for expression of tumor antigen	

Intrinsic regulatory mechanisms of T cells		Use of PD-1 switch receptors to blunt inhibitory effect of PD-1 signaling	(25–34)
		Blocking inhibitory immune receptors to augment adoptive T cell transfer	
		Gene-editing of CAR T cells to disrupt expression of inhibitory receptors	
		Use of CAR T cells overexpressing antiapoptotic proteins	
		Use of CAR T cells downregulating apoptotic proteins	
		Use of dominant negative TGF-β receptor	
		Use of drug/radio resistant CAR T cells	
		Use of more persistent T cells	
		Use of gene edited T cells	

### Tumor Antigen Expression and Heterogeneity

A primary challenge in developing CAR T cell therapy is identifying a tumor antigen that can be targeted safely and effectively [reviewed in Ref. (35)]. Ideally, CAR T cell therapy should target a tumor-restricted antigen to avoid the risk of “on-target/off-tumor” toxicity that may result in an immune reaction against healthy tissues, and at least two criteria should be considered (36). First, the proposed TAA should be differentially expressed on tumor cells compared with essential normal tissue. The CAR response is highly specific and can potentially bind to antigens even at low expression levels in normal tissues. Fine-tuning CAR design to recognize differential expression of antigens on tumor cells continues to evolve, and this represents a dynamic area of research aimed to expand the reach of CAR T cell therapy. Second, the TAA should be broadly expressed on the majority of tumor cells, as the success of CAR T cell therapy is largely dependent on expression of antigens on tumor cells (8, 36). Many of the TAAs identified [e.g., EGFR/EGFRvIII, IL13Rα2, HER2, CD171, mesothelin (MSLN), GD2, and carcinoembryonic antigen (CEA)] are expressed by a wide range of solid tumors, and this affords opportunity for combination therapies using CARs targeting multiple antigens. Table 2 lists antigens that have served as targets for solid-tumor T cell therapeutic studies to date.

**Table 2:** A summary of solid tumor antigens being targeted using CAR T cell therapy.

Antigen	Type of cancer	Endomains	Gene transfer method	Reference
CD171	Recurrent/refractory neuroblastoma	CD3ζ	Electroporation	(37)
EGFRvIII	Glioma	CD28+CD3ζ, 4-1BB	Gamma-retrovirus	(38)
Epidermal growth factor receptor	Gastric cancer	–	Gamma-retrovirus	(39)
Carbonic anhydrase IX	Metastatic renal cell carcinoma	FcRγ	Gamma-retrovirus	(40)
α-folate receptor	Ovarian	FcRγ	Gamma-retrovirus	(41)
HER2	Sarcoma	CD28-CD3ζ	Gamma-retrovirus	(42)
HER2	Glioblastoma	CD28-CD3ζ	pigyBac	(43)
HER2	Osteosarcoma	CD28-CD3ζ	SFG retroviral	(44)
αHER2/CD3	Gastric cancer	CD28-CD3ζ	Gamma-retrovirus	(45)
Carcinoembryonic antigen	Liver metastases	CD28-CD3ζ	Gamma-retrovirus	(46)
IL13Rα2	Glioblastoma	CD3ζ	Electroporation	(47)
IL13Rα2	Glioblastoma	4-1BB, CD3ζ	Lentivirus	NEJM
HER2	Metastatic colon cancer	4-1BB, CD28, CD3ζ	Gamma-retrovirus	(48)
GD2	Neuroblastoma	CD3ζ	Gamma-retrovirus	(49)
GD2	Neuroblastoma	CD28, CD3ζ, OX40	SFG retroviral	(50)
ErbB2 + MUC1	Breast cancer	CD28, CD3ζ	SFG retroviral	(51)
Vascular endothelial growth factor receptor 2 + gp100 + TRP-1 + or TRP-2	Melanoma	–	Gamma-retrovirus	(24)
FAP	Colon and ovarian cancer	CD8α, CD3ζ, 4-1BB	Gamma-retrovirus	(17)
HER2 + CD19	Medulloblastoma	CD28 + CD3ζ	SFG retroviral	(23)
Mesothelin (MSLN)	Malignant Pleural Mesothelioma	CD3ζ and 4-1BB	Lentiviral	(22)
NKG2D	Breast cancer	CD28 + CD3ζ	Gamma-retrovirus	(21)
MSLN	Pancreatic cancer	CD3ζ and 4-1BB	Gamma-retrovirus	(8)
MSLN	Malignant pleural mesothelioma	CD3ζ and 4-1BB	Gamma-retrovirus	(8)

The epidermal growth factor receptor (EGFR) is a trans-membrane receptor tyrosine kinase (170 kDa) that belongs to ErbB oncogene family (52–54). A wide range of normal and tumor cells express EGFR, and deregulation of EGFR is associated with epithelial tumors, such as pancreatic cancer, lung cancer, head and neck squamous cell carcinoma, colorectal cancer, and breast cancer (55, 56). Upregulation of EGFR is associated with poor prognosis in clinical settings (57, 58). Many studies have reported genomic alterations of *EGFR* in glioblastomas affecting both the extracellular and intracellular domains (59, 60). As the most common oncogenic EGFR mutant, with expression on ~30% of glioma cells (60, 61), EGFRvIII contains a deletion of extracellular amino acids 6–273 (62, 63), resulting in constitutive tyrosine kinase activity that promotes aggressive growth and tumor metastasis (64–66). This mutated extracellular EGFRvIII domain presents a tumor-specific, immunogenic epitope for CAR targeting (67, 68). Researchers have evaluated EGFRvIII-CARs for immunotherapy of glioma (38, 68), with the targeting domain derived from EGFRvIII-specific monoclonal antibodies. EGFRvIII-CAR T cells produced interferon-γ, effector cytokines, and were able to kill EGFRvIII+ tumor cells, demonstrating that EGFRvIII-CAR T cells can eliminate glioma cells (38, 67, 68).

Another promising target for brain malignancy is IL13 receptor α2 (IL13Rα2), a monomeric high affinity IL-13 receptor that is overexpressed in the majority of glioblastoma tumors and not expressed at significant levels on normal brain tissue (69, 70). In addition, IL13Rα2 expression is a prognostic indicator of poor patient survival (70). This disease-associated expression profile supports the development of CAR T cells targeting IL13Rα2 for the treatment of glioblastoma and possibly other solid tumors (71). To target IL13Rα2 both antibody- and ligand-based CARs are being evaluated. Our group and others have developed ligand-based CARs utilizing membrane bound IL13 muteins for preferential recognition of IL13Rα2 over the more ubiquitously expressed IL13Rα1 (71). Ligand-based CARs represent a novel class of CAR design. City of Hope is currently in clinical trial evaluating an IL13-ligand CAR T cell platform, and early findings suggest encouraging evidence for safety and therapeutic bioactivity (47, 72).

HER2, a trans-membrane glycoprotein belonging to the EGFR family, is another attractive target antigen for cancer immunotherapy (73, 74). HER2 is overexpressed in osteosarcoma, medulloblastoma, glioblastoma, and ovarian and breast cancer, among others (75–78). Several studies point to the critical role of HER2 in various cancer pathological processes (79), and HER2 overexpression is associated with poor clinical outcomes (80, 81). Ahmed et al. evaluated HER2-CAR T cell therapy for medulloblastoma (78), demonstrating that HER2-CAR T cells are able to target and kill HER2+ medulloblastoma cells *in vitro* and in an established medulloblastoma orthotopic xenogeneic SCID mouse model (78). The researchers reported in a study of osteosarcoma that HER2-CAR T cells, proliferated, produced immunostimulatory T helper 1 (Th1) cytokines, and killed HER2+ osteosarcoma cells *in vitro*, and HER2-CAR T cells caused regression of established osteosarcoma xenografts in locoregional as well as metastatic mouse models (44).

Mesothelin is a tumor differentiation antigen (40 kDa) that is normally present on the mesothelial cells of pleura, peritoneum, and pericardium (82, 83) and is highly expressed in many human cancers, including malignant mesothelioma, pancreatic, ovarian, and lung adenocarcinoma (84–87). MSLN overexpression is associated with proliferation of tumor cells, invasion, and poor survival rates of patients (88–90). The limited expression in normal tissues and high expression in many cancers renders MSLN a potential CAR T cell target (86). Riese et al. evaluated MSLN-CAR T cell treatment for thymoma in a mouse model (91) using a novel strategy designed to improve T cell function by eliminating negative regulators. Given that CAR signaling derives from TCR intracellular domains that function to initiate signal transduction, deletion of negative regulators may augment CAR signaling and effector T cell function. The researchers examined CAR activity in T cells that lacked one or both isoforms of diacylglycerol (DAG) kinase (dgk), normally highly expressed in T cells. The enzymes dgkα and dgkζ metabolize the second messenger DAG and limit Ras/ERK activation. The researchers found that, similar to pharmacologic inhibition of dgk enzymes, dgk-deficient CAR T cells were more effective in limiting the growth of implanted tumors, both concurrent with and after establishment of the tumor. These results indicate that modification of CAR T cells (herein, deletion of negative regulators of TCR signaling) could improve the activity and function of CAR T cells in a solid tumor model. This work highlights the importance of CAR T cell modifications that extend beyond the CAR molecule to T cell-specific functional machinery—modifications that may broaden clinical use and improve the efficacy of CAR T cells (91).

Many preclinical studies of CAR T cells that target stroma and/or TAAs in solid tumor models have evaluated such targets as carbonic anhydrase IX (CAIX), GD2, vascular endothelial growth factor receptor 2 (VEGFR2), folate receptor alpha (FR-α), FAP, and CEA. Several platforms have advanced to the clinic, and Table 3 lists clinical trials corresponding to solid-tumor targets, many of which used first-generation CARs. For example, Lamers et al. assessed first-generation CAIX–CAR T cell therapy in renal carcinoma patients and observed “on-target/off-tumor” side effects and a low persistence of CAR T cells, possibly due to host immune response against CARs (92, 93). Other studies have reported low persistence of first-generation CD171–CAR T cells in neuroblastoma patients and FR-α-CAR T cells in ovarian cancer patients (37, 41). Although results from first-generation CAR T cell therapy trials were disappointing (37, 92, 93), the studies provided data and insights on CAR optimization, leading to the generation of second- and third-generation CARs that may overcome some of the challenges in solid tumor therapy.

**Table 3:** Various clinical trials using CAR T cell therapy in solid tumors.

Type of cancer	Antigen	Identifier	Phase	Status
Glioblastoma	Epidermal growth factor receptor (EGFR)	NCT02331693	I	Recruiting
	EGFRvIII	NCT02844062	I	Recruiting
	EGFRvIII	NCT01454596	I/II	Recruiting
	EGFRvIII	NCT02209376	I	Recruiting
	EGFRvIII	NCT02664363	I	Not yet recruiting
	IL13Rα2	NCT00730613	I	Completed
	IL13Rα2	NCT01082926	I	Completed
	IL13Rα2	NCT02208362	I	Recruiting
	HER2	NCT02442297	I	Recruiting
	HER2	NCT01109095	I	Active, not recruiting

Pancreatic	Mesothelin (MSLN)	NCT02959151	I/II	Recruiting
	MSLN	NCT02465983	I	Active not recruiting
	MSLN	NCT02706782	I	Recruiting

Breast	HER2	NCT02547961	I/II	Recruiting
	MSLN	NCT02792114	I	Recruiting

HER2-positive cancer	HER2	NCT00889954	I	Active, not recruiting

HER2-positive sarcoma	HER2	NCT00924287	I/II	Completed
	HER2	NCT00902044	I/II	Completed

MSLN-positive tumors	MSLN	NCT02930993	I	Recruiting
	MSLN	NCT02159716	I	Active, not recruiting
	MSLN	NCT02590747	I	Recruiting
	MSLN	NCT01583686	I/II	Recruiting

Neuroblastoma	GD2	NCT00085930	I	Completed
	GD2	NCT02107963	I	Completed

CD133-positive malignancies	CD131	NCT02541370	I	Recruiting

Malignant pleural mesothelioma	FAP	NCT01722149	I	Recruiting

Liver metastases	Carcinoembryonic antigen (CEA)	NCT01373047	I	Completed

Pancreatic ductal adenocarcinoma	MSLN	NCT01897415	I	Active, not recruiting

Pleural mesothelioma	MSLN	NCT01355965	I	Completed

Gastric cancer	HER2	NCT02713984	I/II	Recruiting
	HER2	NCT01935843	I/II	Recruiting
	CEA	NCT02349724	I	Recruiting
	CEA	NCT01723306	II	Recruiting

A large number of surface antigens are variably expressed by tumor cells, and among barriers associated with solid-tumor CAR T cell therapy, cell surface antigen heterogeneity features prominently in failures to achieve durable responses (36). O’Rourke et al. found heterogeneity of EGFRvIII expression to be a major barrier in targeting it as a single antigen (67). In a clinical study of EGFRvIII–CAR T cell therapy for glioblastoma, they noted wide regional variation of EGFRvIII expression in tumor samples after EGFRvIII–CAR T cell infusion. Most subjects had loss or decreased expression of EGFRvIII in tumors despite no change in the degree of EGFR amplification or other tumor mutations. The study poses the question whether targeting EGFRvIII alone can provide durable clinical benefits or whether antigen escape will negate the clinical impact. CAR T cell targeting of the tumor antigen IL13Rα2 in patients with glioblastoma has encountered similar hurdles with antigen heterogeneity. Brown et al. reported that treatment with IL13Rα2–CAR T cells mediated a CR in one patient, but the disease eventually recurred 228 days after the first CAR T cell treatment at sites distinct and nonadjacent to the original tumors. Preliminary results suggest the cause of recurrence is decreased expression of IL13Rα2 (72). These and many other studies point to the importance of target antigen overexpression and distribution on most, if not all, tumor cells. As a criterion for patient enrollment on CAR T cell therapy trials, prescreening for the intensity and percentage of target antigen expression on tumor cells by immunohistochemistry and/or immunofluorescent techniques may be predictive of response (17, 23, 24, 51).

Targeting of multiple tumor antigens simultaneously or in a combinatorial strategy could lead to better “killing coverage” and potentially block the emergence of target antigen-null tumor cells (17, 23, 24, 51). Preclinical experiments with trivalent CAR T cells co-targeting HER2, IL13Rα2, and EphA2 showed promise in overcoming glioblastoma variability (94). Analysis of primary glioblastoma patient samples demonstrated the trivalent CARs captured nearly 100% of tumor cells in most tumors and exhibited improved cytotoxicity and cytokine release over monospecific and bispecific CAR T cells. Treatment with the multi-specific CAR T cells *in vivo* controlled established autologous glioblastoma patient-derived xenografts and improved survival of treated animals (94). Another study of dual-targeted CAR T cells specific for MUC1 and ErbB2 demonstrated their effectiveness against solid tumors, particularly breast cancer (51). Proliferation of the dual MUC1/ErbB2 CAR T cells required coexpression of MUC1 and ErbB2 on target tumor cells, and the CAR T cells were effective in killing ErbB2(+) tumor cells. These findings suggest that multivalent CARs may be an effective strategy to box-in heterogeneous tumors and thereby block resistance through tumor escape (51). However, tumor antigen expression loss in glioblastoma patients following CAR T cell therapy specific to one antigen implies that selection of clonal variants resistant to treatment occurs. With the integration of multivalent targets, there may be potential for further selection and the development of treatment resistance over time.

### The Suppressive Solid Tumor Microenvironment

Clinical and preclinical studies have shown that reversing immune inhibitory pathways triggered in many cancers may require CAR T cell modifications beyond the inclusion of co-stimulatory signaling. In contrast to certain blood cancers that have responded well to CAR T cell therapy, solid tumors not only lack conventional co-stimulatory molecules, which are expressed on malignant and normal B lymphocyte targets in hematological malignancies, but also have evolved mechanisms to actively suppress the immune system (95, 96). A number of immunosuppressive pathways can limit the full potential of adoptive CAR T cell therapy. Inhibitory immune receptors are often expressed on T cells following persistent tumor antigen encounter, and these include T-cell membrane protein-3 (TIM-3), lymphocyte-activation protein-3 (LAG-3), T cell Ig and ITIM domain (TIGIT), cytotoxic T lymphocyte-associated antigen 4 (CTLA-4), and programmed death-1 (PD-1). The upregulation of these receptors limit the persistence and activity of the antitumor response of CAR T cells (36).

Tumors employ multiple tactics to evade or misdirect tumor-specific immune response. Many soluble factors that suppress antitumor immune responses have been identified in tissue extracts, serum, and ascites fluid of cancer patients. Tumor cells and macrophages express prostaglandin E2 (PGE2), a soluble factor derived from arachidonic acid and produced by inducible cyclo-oxygenase 2 enzyme (8, 36) that exerts its immunosuppressive effect through subversion of CD8 differentiation, suppression of T cell proliferation, and inhibition of CD4 T cell helper functions (97). The PGE2/EP2/protein kinase A (PKA) signaling pathway mediates immunosuppression through PGE2 (98), which in combination with adenosine activates PKA and blocks TCR activation. A small peptide called the “regulatory subunit I anchoring disruptor” (RIAD) dampens the negative effects of PKA on TCR activation—a function that researchers leveraged to improve T cell function. Through generation of CAR T cells expressing RIAD, Albelda and colleagues showed that inhibition of upstream immunosuppressive mediators of PKA activation such as PGE2 and adenosine could lead to increased TCR signaling, more cytokine release, and increased CAR T cell infiltration, leading to enhanced tumor cells killing (19). Increased inflammatory activity is a hallmark of the tumor microenvironment and creates an abundance of reactive oxygen species (ROS) that substantially impair antitumor activity. Ligtenberg and colleagues hypothesized that CAR T cells coexpressing catalase (CAT) would perform better than regular CAR T cells. They showed that CAT–CAR T cells produced more intracellular catalase, leading to a reduced oxidative state with less ROS accumulation in both the basal and activated states. The CAT–CAR T cells maintained antitumor activity despite an inhospitable environment of high H_2_O_2_ (99).

**Figure 1:**
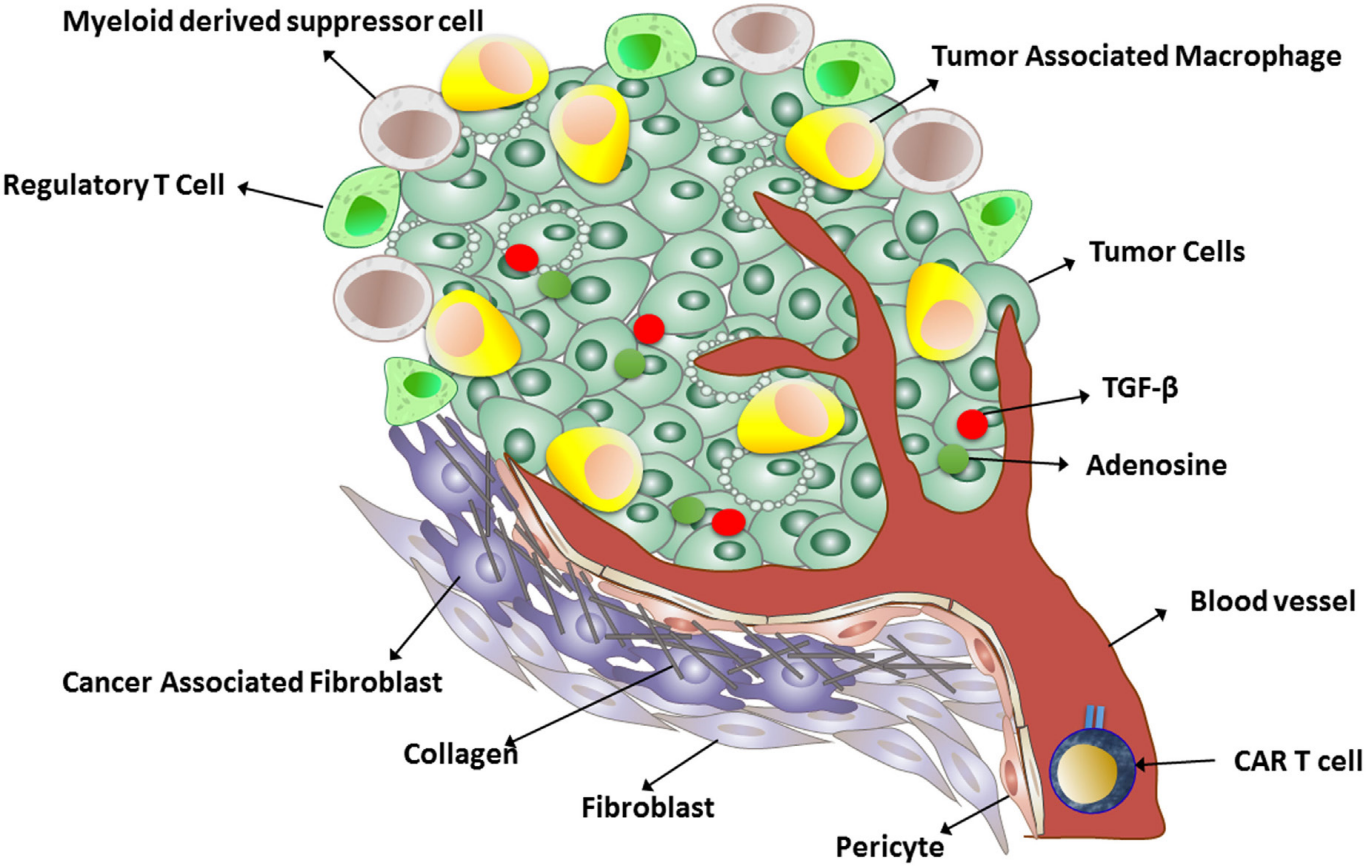
A schematic representation of the immunosuppressive tumor microenvironment.

Many tumors produce transforming growth factor beta (TGFβ), which inhibits T cell activation, proliferation, and cytotoxicity (Figure 1). Chou et al. demonstrated that cell-intrinsic abrogation of TGFβ signaling can enhance T cell persistence and function in a murine model of autochthonous prostate cancer. Moreover, it has been shown that T cells rendered insensitive to TGFβ by transduction with TGFβ dominant negative receptor II were highly effective in eliminating established melanoma-bearing mice (100). This idea has been translated to clinical studies of tumor-infiltrating lymphocytes engineered to express a TGFβ1-dominant negative transgene (NCT01955460). This approach offers an alternative to therapeutic anti-TGFβ monoclonal antibodies (fresolimumab/GC1008).

The recent development of checkpoint inhibitors such as ipilimumab targeting CTLA-4 and nivolumab targeting PD-1 provide further opportunities to enhance antitumor immune response with the potential to produce durable clinical responses though opportunistic autoimmunity (36). John et al. demonstrated that combining CAR T cells and PD-1 blocking antibodies could potently enhance CAR T cell therapy. They found that such combination can significantly decrease the percentage of Gr1+ CD11b+ myeloid-derived suppressor cells, and this was strongly correlated with therapeutic responses in established disease (9). Alternatively, CAR T cells can be further modified intrinsically to enhance their function particularly in the context of the targeted tumor microenvironment. The clustered regularly interspaced short palindromic repeats (CRISPR)/CRISPR-associated (Cas) protein 9 system provides a robust and multiplexable genome editing tool, enabling researchers to precisely engineer specific genomic sequences. The CRISPR/Cas9 system and the simpler Cas9/sgRNA system enable the efficient construction of knockout alleles through the induction of frameshift mutations. With this gene-editing tool, it is possible to generate knock out PD-1 and/or CTLA-4 CAR T cells. This strategy not only prevents potential toxicity of anti-PD-1 or CTLA-4 administration (e.g., opportunistic autoimmunity) but it also would not interfere with normal homeostatic functions of these molecules within the body. Table 1 illustrates various challenges faced by CAR T cell therapy in solid tumors.

### Immune Stimulatory CAR T Cell Modifications

T cell costimulation mediated by CAR second-generation intracellular domains, such as CD28, CD137, CD27, or OX40, fail to overcome many inhibitory effects, especially once T cell anergy/exhaustion have taken effect. Several proposed countermeasures to immunosuppression may enhance antitumor CAR T cell activity in the solid tumor microenvironment, and these include increasing intrinsic CAR T cell activity *via* intracellular modifications, selecting immature or memory T cell subsets for T cell product manufacturing, and targeting cellular and molecular components of the tumor microenvironment. One promising approach entails the use of immunostimulatory cytokines that may revert or block tumor-associated inhibition and activate adoptively transferred T cells. Strategies that provide high levels of immunostimulatory cytokines locally at the antigen site have demonstrated preclinical and clinical efficacy. Animal models using poorly immunogenic tumors revealed that T cells genetically engineered to produce cytokines like IL-7, Il-15, and Il-12 were effective in eradicating tumors (101, 102). Another approach combines blocking immune suppression simultaneous with enhancing stimulatory cytokine production. To protect CAR T cells from the immunosuppressive cytokine IL-4, Mohammed et al. generated a hybrid cytokine receptor in which an IL-4-receptor exodomain was fused to an IL-7-receptor endodomain (103). Transgenic expression of this molecule in CAR T cells reverted the inhibitory effects of tumor-derived IL-4 and promoted T cell proliferation, resulting in enhanced antitumor activity.

Research has shown that locally produced IL-15 improved CAR T cell expansion and prolonged persistence *in vivo* by increasing the expression of antiapoptotic molecules, such as Bcl-2, through activation of the phosphoinositide 3-kinase signaling pathway (104). Local production of other cytokines, such as IL-7 and IL-12, have shown promising results in preclinical studies and clinical studies. Preclinical models showed that IL-7 (and IL-15) induced expansion of CAR T memory stem cells (CD8+ CD45RA+ CCR7+) with greater antitumor activity, which is mediated by increased resistance to cell death following repetitive exposures to the antigen, and maintenance of their migration ability to secondary lymphoid organs. Some studies also coupled CAR T cells with the constitutive or inducible release of IL-12. CAR T cells expressing IL-12 promote a Th1 immune response, reverse anergy in tumor-infiltrating cells, and inhibit Treg-mediated suppression of antitumor effector functions of T cells. CAR T cell expression of IL-12 also dampened production of immunosuppressive cytokines such as IL-10 and TGF-β by tumor-associated myeloid cells. Koneru et al. showed that IL-12 secreting tumor-targeted chimeric antigen receptor T cells (also known as armored-CAR T cells) could eradicate human ovarian xenografts. The authors showed that IL-12 secreting CAR T cells exhibit enhanced antitumor efficacy as determined by increased survival, prolonged persistence of T cells, and higher systemic IFN-γ (14). However, it should be noted that the authors measured the secretion of human IL-12 (p70) in the serum of CAR T cell-treated SCID-Beige mice [with impaired lymphoid development and reduced NK cell activity but normal macrophage and dendritic cells (DCs)] with established ovarian tumors. Because SCID-Beige mice have normal macrophages and DC populations and these cells are endogenous sources of mouse IL-12 production upon tumor challenge, these mice could in fact produce IL-12 after tumor challenge. Therefore, the total concentration of serum IL-12 may have consisted of both endogenous and exogenous IL-12, and may not have reflected IL-12 solely produced by armored-CAR T cells (105). Pegram and colleagues demonstrated that IL-12 producing CD19-CAR T cells eradicate systemic tumors without the need for prior conditioning. Moreover, they showed that such engineered T cells acquire intrinsic resistance to Treg cell-mediated inhibition (106). Chinnasamy et al. demonstrated that adoptive transfer of syngeneic CAR T cells co-transduced with VEGFR2 and constitutively expressing single-chain IL-12 resulted in the regression of established tumors of different histologies without the need for IL-2 administration. Indeed, the VEGFR2–CAR T cells changed the immunosuppressive tumor environment by altering/reducing both the systemic and the intratumoral CD11b+ Gr1+ myeloid suppressor cell subsets that expressed VEGFR2 (107). Alternatively, CAR T cells can be engineered to express cytokine receptors such as IL-7Ra that drive proliferation in response to endogenous IL-7. Perna et al. showed that IL-7 supports the proliferation and antitumor activity of IL-7Rα expressing CAR-GD2+ EBV-CTLs both *in vitro* and *in vivo* even in the presence of fully functional Tregs (108).

### Tumor Trafficking and Infiltration

Insufficient trafficking of CAR T cells to the tumor site represents another barrier for CAR T cell therapy. Studies have shown that improved migration ability of infused CAR T cells to tumor sites may increase their antitumor immune response (109), and efficiency of adoptively transferred T cells infiltrating the tumor site correlates with clinical responses in patients (110–112). Trafficking to the tumor site requires expression and binding of adhesion receptors on both T cells and the tumor endothelium lining. In addition, CAR T cell chemokine receptors must match the chemokines secreted by tumors (8, 36). Chemokine/receptor mismatch has been shown to account for insufficient tumor localization of T cells. Many human tumors either secrete low levels of chemokines or chemokines for which effector T cells lack receptors. Consequently, adoptively transferred T cells may fail find malignant cells. Peng et al. showed that T cell migration to tumor sites could be improved by overexpression of CXCR2, which recognizes tumor-produced CXCL1 (113). Moon and colleagues demonstrated that overexpression of CCR2b in MSLN-targeted CAR T cells led to a more than 12.5-fold increase in CAR T cell migration to mesothelin+ malignant pleural mesothelioma in mice, resulting in enhanced antitumor effects (22). Di Stasi and colleagues showed that expression of CCR4 on CD30-CAR T cells enhanced the migration of these cells toward Hodgkin’s lymphoma-secreting CCL17 in a xenograft animal model (114). Another study reported that expression of CCR2b on GD2-CAR T cells led to a more than 10-fold increase in CAR T cells homing toward CCL2 secreting neuroblastoma cells (115). A separate study also demonstrated that adoptive transfer of NKG2D-based CAR T cells could recruit and activate endogenous antigen-specific CD4+ and CD8+ T cells at the tumor site in a CXCR3-dependent manner to achieve optimal eradication of ID8 ovarian cancer (116).

To address both insufficient T cell migration and the immunosuppressive milieu of solid tumors, researchers combined CAR T cells with an oncolytic virus harboring the chemokine RANTES and the cytokine IL15. The local administration of biological agents, such as cytokines and oncolytic virus, has been previously translated to the clinic with success (117, 118). The experiments of Nishio et al. showed that the modified oncolytic virus provided a direct lytic effect on infected malignant cells, and it facilitated migration and survival of CAR T cells. They reported that the combination induced a potent, dose-dependent, cytotoxic effect on neuroblastoma tumor cells, while leaving the GD2-CAR T cells unharmed. The intratumoral release of both RANTES and IL15 attracted CAR T cells and supported their local survival, leading to increased overall survival of tumor-bearing mice (119). Together these studies suggest that CAR T cell modifications may enhance the efficacy and homing capabilities of adoptively transferred T cells.

Another strategy to increase CAR T cells at the solid tumor site is to break down the tumor stroma (Figure 1). In an interesting study, Garuana and colleagues modified CAR T cells to overexpress heparanase enzyme to degrade the main components of the subendothelial basement membrane and the extracellular matrix (ECM), including the heparan sulfate proteoglycans (HSPGs), in order to facilitate CAR T infiltration into tumor stroma. The ECM is an integral component of the stroma, and therefore, T cells attacking stroma-rich solid tumors must be able to degrade HSPGs in order to access tumor cells and exert antitumor effects. The authors found that engineered CAR T cells expressing heparanase showed improved capacity to degrade the ECM and promoted T cell infiltration and antitumor activity (50). The studies support the concept that generation of CAR T cells with a chemokine receptor or enzyme could facilitate infiltration into the tumor stroma and enhance antitumor efficacy. Although these approaches have been shown to be effective in animal models, introducing chemokine receptor transgenes into CAR T cells for adoptive cell therapy has yet to be tested in humans.

An entirely different approach to promote homing of CAR T cells to solid tumor sites involves delivery of CAR T cells directly to the tumor site, a departure from the more common intravenous (i.v.) route of administration. Adusumilli and colleagues showed that compared to i.v. administration, local CAR T cell administration resulted in greater T cell antitumor potency with reduced T cell doses, partially due to early CD4+ T cell activation and the systemic benefits that ensued (20). Brown et al. described promising results of locoregional CAR T cell delivery for the treatment of glioblastoma (72). A comparison in one patient of two intracranial CAR T cell delivery routes—infusion into the resected tumor cavity and infusion into the ventricular system—pointed to the potential impact of the route of administration. In this patient, both routes (intracavitary and intraventricular) had low toxicity profiles but differed in subsequent tumor growth at distant sites. While intracavitary therapy appeared to control local tumor recurrence, glioblastoma progressed at distant sites, including the onset of new lesions. By contrast, after intraventricular administration of CAR T cells, regression of all central nervous system tumors, including spinal tumors, was achieved (72).

## Clinical Studies—Looking Ahead

In early clinical studies with first-generation CAR T cells, therapeutic T cells showed little persistence, so the efficacy and safety were difficult to assess. Although targeting solid tumors is still in the early stages, trials have already shown antitumor activity in solid tumors such as neuroblastoma. Louis et al. developed CAR T cells targeted to the validated tumor antigen GD2, for which the safety of monoclonal antibody therapy was previously demonstrated (49, 120). As one of the first CAR T cell therapy trials, the first-generation GD2-CAR T cells were administered to children with advanced neuroblastoma, with 3 of 11 patients experiencing CRs, no substantial toxicity observed, and sustained clinical benefit for several patients reported (49, 121). The results are especially encouraging in light of CAR T cell advances that incorporate co-stimulatory signaling motifs in addition to CD3ζ, as was used in this trial. Unlike the favorable safety profile observed with GD2-CAR T cells, another CAR T cell trial in a patient with colon cancer metastatic to the lungs and liver resulted in death of the patient. The CAR targeting domain was based on the humanized monoclonal antibody trastuzumab (herceptin), specific to the tumor antigen Her2. The outcome was attributed to Her2 expression on normal lung and/or cardiac tissue (49). Importantly, this trial administered substantially higher numbers of CAR T cells than most other trials, raising the question of whether lower doses of HER2-CAR T cells might be safe. One takeaway from this experience is that antigens safely targeted by monoclonal antibody therapy cannot be assumed safe for CAR T cell therapy.

Glioblastoma is the most common and most malignant of brain tumors. It grows aggressively in the CNS and no current treatment is curative. CAR T cell preclinical work has shown promise, and current CAR T cell clinical trials in glioblastoma target three different antigens, EGFRvIII, HER2, and IL13Rα2 (67, 72, 78). O’Rourke et al., reported on 10 patients with glioblastoma who were treated with EGFRvIII-CAR T cell therapy. Manufacturing CAR T cells from patients with recurrent GBM was feasible, and no cross reactivity of EGFRvIII-CAR T cells with wild-type EGFR was observed. However, clinical benefit was indeterminate because treatment-related changes common to immunotherapy such as inflammation were difficult to distinguish by imaging from tumor progression. The research team did observe that the i.v.-infused CAR T cells trafficked to the brain and demonstrated antigen-specific activity. Two barriers to therapy were clear from the study: heterogeneity of EGFRvIII expression, as described earlier, and the immunosuppressive tumor microenvironment, which intensified upon CAR T cell administration. An increase in non-CAR polyclonal T cells was observed in the tumor environment, which phenotypic analysis indicated to be comprised mostly of immunosuppressive regulatory T cells based on their expression of CD4, CD25, and FoxP3. In addition, immunosuppressive molecules such as IDO1, PD-L1, and IL-10 were upregulated after CAR T cell infusion. These findings suggest that EGFRvIII-CAR T cells induced an immunosuppressive response, and that measures to counter such a response, such as immune checkpoint blockade might work synergistically with CAR T cell therapy.

Safety concerns over targeting HER2 with CAR T cells were raised by the death of a patient who had received third-generation HER2-CAR T cells (10^10^ cells) after lymphodepleting chemotherapy, as described above. Ahmed et al., developed a second-generation HER2-CAR, and in patients with sarcoma, CAR T cell treatment (up to 10^8^/m^2^ cells) demonstrated no evident toxic effects, some indicators of antitumor activity, but limited T-cell persistence. To optimize the persistence of adoptively transferred T cells, the team engineered CARs into virus-specific T cells (121) in which costimulation results from native TCR (αβTCR) engagement with latent virus antigens on professional antigen-presenting cells. The group has established the safety of adoptively transferred polyclonal virus-specific T cell lines, enriched for cytomegalovirus, Epstein–Barr virus, and adenovirus, in hematopoietic stem cell transplant recipients. A phase 1 dose-escalation study established the safety of autologous HER2-CAR virus-specific T cells in 17 patients with progressive glioblastoma. Although the CAR T cells did not expand, they were detectable in the peripheral blood for up to 12 months. Of eight patients, one had a partial response and seven had stable disease. The median OS was 11.1 months after T cell infusion and 24.5 months after diagnosis. The results highlight the need for improvement in expansion, function, and persistence of the HER2–CAR T cells. Manipulations of the immune system to thwart immunosuppression and/or targeting multiple antigens to overcome glioblastoma heterogeneity may improve response rates and outcomes. Preconditioning regimens, such as lymphodepletion, may aid in increasing T cell responses. However, more work is needed to delineate how these treatments can augment CAR T cell therapy.

A clinical trial of autologous CAR T cells targeting IL13Rα2 provided the first evidence for a CAR T cell−mediated CR to therapy in glioblastoma. After receiving IL13Rα2-CAR T cell therapy, a patient with recurrent multifocal glioblastoma experienced dramatic improvements in his quality of life, including the discontinuation of systemic glucocorticoids and a return to normal life activities. Notable in this case was the potential role of the endogenous immune system in the antitumor responses. Immediate increases in endogenous immune cells and inflammatory cytokines after each intraventricular infusion of CAR T cells may have reflected recruitment and stimulation of the host immune system and may explain how a CR was achieved despite non uniform expression of IL13Rα2 on the tumors. After each intraventricular infusion of CAR T cells, rapid and pleiotropic changes in levels of inflammatory cytokines in the cerebrospinal fluid were observed, with significant increases in the interferon-γ-inducible chemokines CXCL9 and CXCL10, which have antitumor potential but did not affect neurologic function or the general well-being of the patient. This clinical experience, along with the studies of EGFRvIII-CAR T cells and HER2-CAR T cells against glioblastoma provide initial evidence of the safety and antitumor activity of CAR T cell immunotherapy in patients with malignant brain tumors.

## Concluding Remarks

New generations of optimized CARs could contribute to improved clinical responses, as could the combination of CAR T cells with other immunotherapeutic modalities such as checkpoint inhibitors, oncolytic viruses, vaccines, or cytokines, which may synergistically enhance therapeutic efficacy in solid tumors.

The generation and optimization of engineered cells derived from different cell populations offers other immunotherapeutic avenues. To date most studies employ T cells bearing αβ receptors, but γδ T cells possess a combination of innate and adaptive immune properties that may be conducive to cancer immunotherapy (122–124). Studies show that γδ T cells play a key role in tumor immunosurveillance and antitumor immune responses (124–126). In contrast to T cells bearing αβ receptors, γδ T cells are not susceptible to antigen processing and presentation defects, which is one strategy for cancer immune evasion. Moreover, the absence of co-stimulatory molecules resulted in the appearance of tumor clone(s) resistant to αβ (but not γδ) T cell-mediated cytotoxicity. Further, γδ T cells are able to directly lyse stressed cells (e.g., malignant transformations), produce a range of inflammatory cytokines and chemokines, present antigen to αβ T cells (i.e., T cell priming), and induce DC maturation (127, 128). Another favorable characteristic of γδ T cells is the migration of specific subsets to mucosal epithelial surfaces. This could be a crucial factor for successful immune or tumor-surveillance functions. Tissue-specific trafficking of γδ T cells to epithelial tissues as well as to tumors originating from such tissues has important implications for the design of unique immunotherapeutic strategies (129–132). As noted above, one of the potential challenges of adoptive T cell therapy is insufficient trafficking of effector T cells to tumor sites. Inherent γδ T cell tropism to epithelia tissues may overcome the barrier of insufficient trafficking of effector T cells to epithelial solid tumor sites. Moreover, expression of NKG2D ligands on tumor cells derived from these tissues can enhance the antitumor activity of the adoptively transferred T cells, potentially acting synergistically with CAR stimulation and reducing the likelihood of immune escape through antigen loss. Taken together, many properties of γδ T cells make them an attractive candidate platform for CAR T cell therapy for solid tumors.

Encouraging results in which CAR T cells mediate robust antitumor responses have been observed in certain blood cancers as well as isolated cases of patients with solid tumors, but engineered T cells have yet to yield high response rates responses in patients with solid tumors. Better understanding of the various solid tumor features that are problematic for adoptive T cell therapy will guide the development of new generations of T cells that may prove more effective in overcoming the challenges of solid tumor malignancies.

## Author Contributions

Conception and design of paper was done by CB and BB. Initial draft was completed by HM. Editing and further drafts were done by AR and JS. All authors reviewed final version of paper.

## Conflict of Interest Statement

Patents associated with CAR design, T cell manufacturing, and delivery have been licensed by Mustang Bio., Inc., for which CB and BB receive licensing and consulting payments. The remaining authors declare that the research was conducted in the absence of any commercial or financial relationships that could be construed as a potential conflict of interest.
