# Corrigendum: Chimeric Antigen Receptors T Cell Therapy in Solid Tumor: Challenges and Clinical Applications

**DOI:** 10.3389/fimmu.2019.00780

**Published:** 2019-04-17

**Authors:** Hamid R. Mirzaei, Analiz Rodriguez, Jennifer Shepphird, Christine E. Brown, Behnam Badie

**Affiliations:** ^1^Department of Medical Immunology, School of Medicine, Tehran University of Medical Sciences, Tehran, Iran; ^2^Division of Neurosurgery, Department of Surgery, City of Hope National Medical Center, Duarte, CA, United States; ^3^Department of Hematology and Hematopoietic Cell Transplantation, T Cell Therapeutics Research Laboratory, City of Hope Beckman Research Institute, Duarte, CA, United States

**Keywords:** immunotherapy, T cell therapy, chimeric antigen receptor, CAR, solid tumors

In the original article, there was an error. We neglected to disclose a conflict of interest.

A correction has been made to the **Conflict of Interest statement**:

“Patents associated with CAR design, T cell manufacturing, and delivery have been licensed by Mustang Bio., Inc., for which CB and BB receive licensing and consulting payments.”

The authors apologize for this error and state that this does not change the scientific conclusions of the article in any way. The original article has been updated.

